# Editorial

**Published:** 1872-06

**Authors:** 


### EDITORIAL.

### Celluloid.

Since the introduction of this material as a base for artificial dentures, there has been a very general disposition to test it, and, as usual with such things, with quite diverse results; there is sufficient cause for this, aside from the nature of the material itself. It is irksome to subject one's self to a tedious course of experimental training, when there is something at hand with which there is entire familiarity, and the desired results can be obtained easily. The difficulties in the use of hard rubber for artificial dentures, which at first were very great, have been so completely overcome that every one now works it as readily as the molder forms bricks from clay.

Upon the introduction of rubber for dentures so many and so great were the difficulties attending its manipulation, that years of patient effort and toil were required to overcome them all. Even those best skilled in its use made failures. The success in the use of celluloid is certainly more promising at this period of its use, than was rubber at the same time; and to those who desire to avoid the use of rubber, and must have something for cheap dentures, we would suggest, be patient with celluloid; become familiar with its nature and the proper method of working it. In the construction of artificial dentures there are certain processes and manipulations that are common to all styles; for example, obtaining models, and making a good adaptation of the plate to the model, the selecting, adapting and arranging the teeth; and in reference to the teeth for celluloid, there seems to be no occasion for gum teeth, either single or in blocks; plain teeth in all cases being the best; as the celluloid is quite adapted in color and in every other respect for the gum restoration, and the adaptation and adjustment of the material to the teeth is more easily secured, and is more perfect.

The teeth are ground to a base plate of wax or gutta percha, and flasked in the same manner as for rubber.

Directions for using are furnished with the celluloid material that are in the main correct, though in a few particulars it is found better to deviate a little from them. For instance, it is better to raise the heat to about 310° before bringing the flasks together at all, then it will go together, all the previous steps having been made, very easily, and without any liability of injuring the model, or disarranging the teeth. After the flask is brought together, it is better to let it remain in the oil at 310° for ten or fifteen minutes; this seems to give toughness and a better body, and renders any after change less liable.

In concluding these remarks, we take pleasure in quoting a few remarks by Dr. A. C. Ford, in the *Dental Cosmos*, of April, 1872. He says:

"In manipulating celluloid, I have no idea that those who employ water will ever succeed (though I may be mistaken), as I consider it takes a greater heat than 212° to thoroughly soften the material. I do not commence to close the flask (*i. e.* with the clamp) until the thermometer indicates the oil in at 260°, and after the flask is brought thoroughly down, I keep it in the oil at 310° for from five to ten minutes; this, I think, helps to toughen or rather mature the base. I do not think the oil should be used more than twice, and I think better results are obtained by using fresh oil every time. I would, however, be glad to hear from some who have been working in this base more extensively; but I do not desire the opinion of any except those who strive to attain the highest results. Some may assert that the celluloid will not warp as long as it is worn continually in the mouth, but that if left out for a few days it will be found to have altered its shape (this was said of pyroxyline, how true I know not); but I have caused it to pass this ordeal also, for lately, having been indisposed and confined to my room, I took out my plate, and kept it out for about four or five days; when I replaced it there was no change.

In addition to the above, I would say that my experience with this material is that it makes a much handsomer plate, is lighter, adheres closer to the teeth, and adapts itself more closely to the mouth, and, *if durable*, I have no hesitstion in asserting it to be decidedly preferable to rubber."

---

Dr. Henry A. Downing, a dentist of Cincinnati, was married yesterday at the Fifteenth-street Christian church, to Miss Mollie E. Shuck, of this city. The nuptials were solemized by Elder J. C. Keith, in the presence of a great number of friends. The newly-wedded pair left immediately for Cincinnati.—*Louisville Courier.*

We welcome the doctor and his estimable bride to our city, and hope that they may have a full measure of the joys and happiness, derivable from the position they have so recently assumed.—ED. REG.

### State Board of Dental Examiners.

The semi-annual session of the Ohio State Board of Dental Examers was held on the 6th and 7th inst., in the rooms of the Ohio Dental College, in Cincinnati, all the members of the Board being present. The following members of the profession presented themselves, were examined, and received certificates of qualifications to practice dentistry, viz:

| | |
|---|---|
| Henry H. Harrison, | Cadiz, Harrison Co., O. |
| Stiles B. Messenger, | Sidney Shelby, Co., O. |
| Wm. H. Hague, | New Vienna, Clinton Co., O. |
| Joseph M. Chambers, | Marietta, Washington Co., O. |
| Frank O. Jacobs, | Coshocton, Coshocton Co., O. |
| Chas. T. Sweet. | Cumberland, Gurnsey Co., O. |
| Wm. J. Downard, | Wapakoneta, Auglaize Co., O. |
| Marcellus Sabin, | Lima, Allen Co., O. |
| Gibbon A. Case, | Kent, Portage. Co., O. |
| Benjamin F. Bowman, | Winchester, Preeble Co., O. |
| Edward E. Rogers, | Hudson, Summit Co., O. |
| J. N. Foote, | Middletown, Butler Co., O. |
| Laird W. Nevius, | Mansfield, Richland Co., O. |
| S. G. Ashcroft, | Sandusky, Erie Co., O. |
| Wm. P. Rice, | Mt. Union, Stark Co., O. |
| Jacob Shisler, | New Garden, Columbia Co., O. |

This work being finished, the Board adjourned to meet in Columbus on the first Monday of December next, at 12 o'clock M. That will be the only session held between this and the time at which the State law regulating dental practice comes into full operation; viz: January 1st 1873. All those practicing in the State without a deploma or certificate at that time will do well to make a note of the fact.

### Northern Ohio Dental Society.

The annual session of the Northern Ohio Dental Society was held at Put-in-Bay on Teusday, May 28. There were thirty members of the profession in attendance. There were several from Southern Michigan. The sessions continued two days and a morning session of the third day. It was a most enjoyable meeting on several accounts. And first, the place is most charming; no one can go there without being delighted with the beautiful prospect and pleasant surroundings. The hotel accommodations furnished by Messrs. Sweney and West are most ample and complete. So many attractions exist at this place, that we think the American Dental Association should hold its meeting of 1873 there. The meeting just held was one of unusual interest. The subjects were interesting, and in their discussion nearly all the members joined.

The propriety of embracing in this society members of the profession from the southern part of Michagan was suggested and very favorably received; definite action however was laid over till the next meeting. We think the time has arrived when all dental societies that have secured a permanent organization should seek to extend and enlarge their sphere of operation and usefulness. Much may be done in this direction by seeking and bringing in new subjects for consideration, any and all that may in any way be made tributary to our profession, and also by presenting old subjects in a new dress. All the older and established societies should pursue such a course as would impose some definite duty upon each and every one of its members. This may be done by dividing the body into sections of from two to four persons each, and then exacting of each section or division the performance of its assigned duty. By such an arrangement vastly more will be accomplished than when no such organization is attempted, and every one is left unaided, to follow his own inclinations, and do something or nothing—too often nothing. We shall watch with interest, the state or local society that first inaugurates some such method. It is clearly within the province of especially all State societies, to make a full and distinct declaration upon matters of general and important interest to the profession. Such as professional education in all its aspects; legal enactments for restraint and encouragement; the organization of local societies, and the holding normal classes for clinical instructions either with or without local societies; and upon professional literature, journalism, etc. These with many other things that might be named come legitimately within the province of our societies.

